# Campbell on Uterine Displacements

**Published:** 1875-08

**Authors:** 


Resume of a Report on Position, Pneumatic Pressure and Mechanical Appliance in Uterine Displacements. Read before the Medical Association of Georgia, April 23d, 1875. By Henry Frazer Campbell, A.M., M.D., etc.

The pamphlet before us embodies the imperfect synopsis of Professor Campbell's Report, published in the June number of this Journal, and also a more ample *resume* from his own pen, setting forth his ideas upon the subject in a clearer and more satisfactory light. The pamphlet is handsomely illustrated by engravings, which give valuable assistance in correctly apprehending the views insisted upon by the writer.

